# Note from the editors: The year 2016 gone, 2017 just started

**DOI:** 10.2807/1560-7917.ES.2017.22.2.30442

**Published:** 2017-01-12

**Authors:** Eurosurveillance editorial team

**Affiliations:** 1 European Centre for Disease Prevention and Control (ECDC), Stockholm, Sweden

At the start of a new year, we provide our readers with a look back over the past 12 months and an outlook into the future. In 2016, *Eurosurveillance*’s 20th anniversary was an opportunity to review the evolution and achievements of the journal so far, and we summarised our personal views in an editorial accompanying a special anniversary print edition of *Eurosurveillance* [1].

For the journal, as well as for infectious diseases specialists and public health experts, Zika virus (ZIKV) remained one of the main topics in 2016. The virus continued to spread, while increasing evidence led to scientific consensus about a causal link between ZIKV infection and occurrence of Guillain–Barré syndrome (GBS) as well as microcephaly and other congenital brain abnormalities [2]. Further, knowledge emerged that ZIKV can be transmitted by routes other than the vector-borne one. In February 2016, when an article in *Eurosurveillance* reported on an autochthonous case of ZIKV disease caused by possible sexual transmission, evidence supporting the possibility of this transmission pathway was still limited [3]. This changed rapidly, and sexual transmission of the virus, even by asymptomatically infected individuals, became the focus of scientific studies and the centre of attention of many public health experts. International and national public health organisations took new findings into account and, whenever necessary, updated advice and guidance on how to prevent sexual transmission [4-6].

Colistin serves as last resort antibiotic for treatment of patients infected with highly resistant, e.g. carbapenem-resistant, Gram-negative bacteria. For public health microbiologists and those concerned with antimicrobial resistance, an important finding in 2016 was the extent of the diffusion of plasmid-mediated colistin resistance, conferred by the *mcr-1* gene. The first findings in China of this new threat to healthcare and patient safety were published in November 2015 [7]. By July 2016, we had published seven articles on detection of the *mcr*-1 gene in Belgium, France, the Netherlands, Portugal and Spain, as well as Brazil and Tunisia [8-14]. Already in March 2016, an editorial in *Eurosurveillance* took stock of the reported evidence of the *mcr-1* gene in food animals, food and humans [15].

In addition, we dedicated special issues to methods and approaches for surveillance, diagnosis and strain typing of *Clostridium difficile* and the impact of anthropogenic changes to water on human pathogens. Influenza vaccine effectiveness, in particular the effectiveness of live attenuated influenza vaccines in children, the emergence and surveillance of enteroviruses (EV) such as of EV D-68 causing severe respiratory illness particularly in children, vaccine preventable diseases, HIV/AIDS and sexually transmitted infections, as well as food- and waterborne outbreaks, were among the variety of other subjects that we covered in 2016.

We published 195 articles (73 rapid communications, 122 regular articles) and a total of 39 editorials, letters and meeting reports. On average there were 72 submissions to *Eurosurveillance* per month. Of the 864 articles we received in 2016, we accepted a little less than 20% for publication.

Articles were selected for publication with the help of our wide network of reviewers. Nearly 500 experts worldwide dedicated their time in 2016 to guide us in the decision-making process by sharing their views and comments on articles. We are grateful to them and published a list with their names in our first 2017 issue [16]. In the choice and commissioning of articles we also rely on our associate editors and editorial advisors. They are our link to academia and public health practice and help us find the right strategy and mix of topics for our audience. We are glad to have them on board and value their contributions. We are also supported by experts, many of them our colleagues at the European Centre for Disease Prevention and Control (ECDC), whose names do not necessarily appear in the reviewer list but who are ready to listen to us and provide us with their feedback and to whom we would like to express our gratitude. We acknowledge the continued funding, logistic support and encouragement from our publisher, ECDC. Together with the editorial independence which ECDC and its Director grant the editor-in-chief, the sustained funding has been a key element in the positive evolution and recognition of the journal over the past decade.

The joint forces of *Eurosurveillance* contributors over two decades have borne fruit. Also in 2016, commonly used indices of ‘impact’ such as the impact factor, SCImago journal rank and Google metrics positioned the journal well among others in the same field, and in a similar fashion to previous years.

For 2017, some changes are envisaged for the journal; the most important one will be the launch of a new website that should go live in the second part of the year. It will provide our audience with more modern features and user-friendly navigation. A minor change for readers of our national bulletin section will be that we will change the frequency of its publication from monthly to quarterly. The first 2017 ‘In the national epidemiological bulletins – a selection from current issues’ will be published in the 30 March issue. In the last quarter of 2016, we had a restricted call for a special issue on Immunisation Information Systems and we envisage publication of the special issue at the end of April. A formal call to provide evidence for screening of infectious diseases in migrants will soon be published.

At the beginning of 2017, we do not know what surprises the year may bring, however, *Eurosurveillance* will remain a platform for the infectious disease and public health community to share findings that help protect and improve public health, and we look forward to receiving such contributions.
